# Effect of an Ad Libitum Milk Supply During the First Three Weeks of Life of Dairy Calves on Heart Rate and Heart Rate Variability During Feeding and Rehousing

**DOI:** 10.3390/vetsci12101009

**Published:** 2025-10-17

**Authors:** Luise Prokop, Gundula Hoffmann, Martin Kaske, Steffi Wiedemann

**Affiliations:** 1 Animal Health, Institute of Animal Breeding and Husbandry, University of Kiel, Olshausenstraße 40, 24098 Kiel, Germany; lprokop@lksh.de; 2 Department Sensors and Modelling, Leibniz Institute for Agricultural Engineering and Bioeconomy, Max-Eyth-Allee 100, 14469 Potsdam, Germany; ghoffmann@atb-potsdam.de; 3 Swiss Bovine Health Service, University Zürich, Winterthurer Strasse 260, 8057 Zürich, Switzerland; martin.kaske@kgd-ssv.ch; 4 Livestock Sciences and Environmental Impacts, Sustainable Food Systems Research Centre, Rhine-Waal University of Applied Sciences, Marie-Curie-Street 1, 47533 Kleve, Germany

**Keywords:** heart rate, heart rate variability, stress, ad libitum feeding, calves

## Abstract

**Simple Summary:**

This study investigated the effect of different milk feeding strategies on the physiological stress response in young calves. Half of the calves were fed a restricted amount of milk twice daily (RES group), while the other half received an unlimited supply (ADL group) during their first three weeks of life. Heart rate (HR) and heart rate variability (HRV) were measured as indicators of stress during feeding and during the rehousing process. The findings suggest that calves on a restricted feeding schedule may exhibit a more pronounced cardiac stress response at feeding time, potentially due to hunger. While both groups showed an expected increase in HR during the stressful event of rehousing, some HRV parameters indicated that calves with an unlimited milk supply may have had a different physiological coping mechanism. These results imply that a more abundant milk diet in early life may influence how calves handle physiological challenges. Further research is needed to fully understand these effects.

**Abstract:**

Early-life feeding strategies are known to affect growth, behavior, and stress physiology in dairy calves. This study examined the effects of different milk feeding regimes on heart rate (HR) and heart rate variability (HRV) during feeding and rehousing as indicators of autonomic activity. Dairy calves were fed either a restrictive milk allowance twice per day (6 L/d; RES; *n* = 21) or an unlimited amount of milk (ad libitum; ADL; *n* = 24) during the first three weeks of life. All calves were housed in individual straw bedded hutches from d 1 to 23 of life and were moved to a group pen on d 23 ± 2 of life. Starting at least one day before rehousing until one hour after the rehousing process HR, HRV, and variables in the time and frequency domain were measured continuously using a portable recording system. To study the cardiac response to the feeding process, six time windows of 5 min each were chosen as follows: resting time at 5.00 a.m., start of personnel activity in the barn, 15 min before feeding, during feeding, 15 min after feeding, and 1 h after feeding. For the evaluation of cardiac response to an unknown stressor such as rehousing, four time windows of 5 min each were selected as follows: resting time at 5.00 a.m., during rehousing, 30 min after rehousing, and 1 h after rehousing. During resting as well as before feeding and rehousing, HR was higher in ADL calves compared with RES calves. During feeding and rehousing, HR reached peak values which were comparable in both groups. HRV variables of the time and frequency domain indicated a shift towards a sympathetic dominance in the balance of the autonomic nervous system during feeding time, particularly in RES calves. Differences between resting and feeding values were demonstrated in RES calves at low-frequency and high-frequency power, whereas no differences were observed in ADL calves which did not react to the feeding process. The cardiac response of calves to rehousing was inconsistent in both groups. An increase in RMSSD and SD1 in ADL calves indicated that the vagal component in the vegetative neurological control was increased in these calves during rehousing. In conclusion, our findings indicate that restrictive milk feeding alters autonomic regulation and may increase physiological stress responses in calves.

## 1. Introduction

Over the past decades, numerous studies have compared the effects of enhanced nutrient supply during early postnatal life with the conventional restrictive feeding regime of calves (typically 10% of body weight/day). It has been shown that ad libitum milk feeding in individual pens can result in intakes of up tof 25% of body weight, comparable to the milk consumption of dam-suckled calves [1,2,3]. Such higher intakes are consistently associated with improved growth during the first weeks of life and generally with beneficial or neutral effects on health and behavior summarized in [4,5].

The extent to which restrictive feeding increases autonomic stress responses around feeding times remains unclear, as calves may experience restlessness due to hunger and unsatisfied sucking motivation. Recent non-invasive monitoring studies have shown that nutritional regimes can affect behavior and physiological indicators such as heart rate variability [6], but the direct role of restrictive milk feeding in shaping acute stress responses still requires clarification. Nutritive sucking is known to be more rewarding and reinforcing than non-nutritive sucking in pre-weaned calves [7].

It is well established that stressors such as transport and heat affect autonomic regulation and heart rate variability in calves [8,9]. Less is known, however, about whether the level of nutrient supply during the first weeks of life influences the stress response to other common challenges, such as rehousing. Rehousing from individual hutches into group pens typically occurs after the first weeks of life and involves close contact with humans, exposure to a novel environment, and interactions with unfamiliar group mates, all of which may result in social stress [10].

Heart rate variability (HRV) has proven to be a reliable method for assessing autonomic nervous system activity in calves [11,12,13,14]. Unlike measures of hypothalamic–pituitary–adrenal (HPA) axis activity, HRV recordings can be collected without human interference, allowing objective and continuous assessment of stress. HRV reflects the balance between sympathetic and parasympathetic branches of the autonomic nervous system, which dynamically regulate physiological responses to both external and internal stimuli to maintain homeostasis. Thus, HRV provides valuable insights into cardiac stress responses to physical, pathological, or emotional challenges [11,12,14]. In newborn calves, resting heart rate typically ranges between about 100 and 140 beats per minute [9,14], providing a physiological context for interpreting HRV measures.

The objective of this study was therefore to investigate HRV in calves before, during, and after (a) feeding and (b) rehousing, in order to clarify the influence of early-life feeding intensity on stress responsiveness.

## 2. Materials and Methods

### 2.1. Calves, Management and Feeding

The study which has been described in detail previously [15] was conducted at a research dairy farm in Schleswig-Holstein, Germany, which housed 190 cows with a mean 305d lactation milk yield of 11,500 kg. Male (*n* = 21; birth weight 43.9 kg) and female (*n* = 24; birth weight 41.4 kg) Holstein–Friesian calves born between November and February were enrolled in the study. The farm was authorized under §11 of the German Animal Welfare Act, and the experiment was carried out in accordance with strict federal and international guidelines on animal research (approval no. AZ 313-72241.123-34, at that time Ministry of Agriculture, Environment and Rural Areas [MLUR], Schleswig-Holstein, Germany). All calves were separated from their dam within the first 1–8 h after birth and moved into individual straw-bedded hutches shortly after birth. Within the first hours of life all calves were fed 3 L colostrum from their dam. Subsequently, calves were randomly allocated to an ad libitum group (ADL, *n* = 21; 9 male and 12 female calves, birth weight 47.8 kg) or a restricted feeding group (RES, *n* = 24; 12 male and 12 female calves, birth weight 42.5 kg). For farm management reasons, assignments were made in blocks of 2–3 animals.

After the supply of colostrum ADL calves received 6 to 9 L milk twice a day (07:00 a.m. and 05:00 p.m.) in nipple buckets. During the morning feeding the rest milk in the nipple bucket was filled up to 6 to 9 L in total and the amount was documented. In the evening residual amounts were weighed, discarded and buckets were washed and refilled. During the first 4 d of life calves received transition milk. From d 5 to d 23 of life a mixture of pasteurized waste milk (Milk Taxi 150 L Pasteurizer; Holm & Laue, Westerrönfeld, Germany) and surplus transition milk was offered. A supplement (Quota Mix, Sprayfo, Sloten GmbH, Diepholz, Germany; CP 11.4%, crude fat 1%, CF 0%, crude ash 8%; 100 g/L milk) and an acid (AcidFit, Sprayfo, Sloten GmbH, Diepholz, Germany; 2.5 g/L milk) was added to the milk mixture. The ingested volumes of milk were documented twice daily for each calf.

After the colostrum feeding the RES calves were offered 3 L transition milk twice per day (07:00 a.m. and 05:00 p.m.) in nipple buckets. From d 5 to d 23 of life 3 L of the supplemented and acidified waste and transition milk mixture which was offered to the ADL calves was fed.

ADL and RES calves were moved into straw-bedded group pens on d 22.7 ± 2 of life. All calves were led by ropes from the individual hutch to the group pen by a familiar person (distance approximately 200 m, average duration 6 min). Typically, one calf was moved at a time. If a new pen was to be used two calves were rehoused in parallel. Maximum number of animals per group was 5. At all times, all calves had free access to water and calf starter.

### 2.2. Body Weight

The body weight of each calf was recorded after birth and right before rehousing (Soehnle Professional, Modell 3025, Soehnle Industrial Solutions GmbH, Backnang, Deutschland, Germany; readability 0.5 kg). Calves were guided on a halter over the scale, which was located directly on the way out of the calf barn with individual hutches.

### 2.3. Heart Rate Variability

Heart rate (HR) and heart rate variability (HRV) were recorded with a portable recording system (eMotion, Mega Electronics, Kuopio, Finland) attached with two self-adhesive skin electrodes (Ambu^®^ Blue Sensor VL, Ballerup, Denmark) to the left side of the calves. The electrodes were positioned next to the heart in height of the elbow and approximately one hand under the withers. To ensure a strong attachment of the electrodes to the skin, the skin was firstly sheared and cleaned with a dry towel and an adhesive spray (Henry Schein, Hamburg, Germany) was applied. The two electrodes were connected via cable to the recording device, which stored the data of the individual calf. A belt was fixed around the thorax of each calf to secure the position and to protect the electrodes against chewing by other calves after rehousing.

The recordings of data were started at least one day before rehousing to allow the calves to adapt to the electrodes and the belt. One hour after rehousing the electrodes and the belt were removed to avoid later chewing by other calves due to increasing exploratory behavior. Cardiac response to feeding was analyzed during morning feeding one day before rehousing in 39 calves of which an exact feeding time was recorded (ADL: 9 male and 10 female calves; RES N = 8 male and 12 female calves). The measurements of the rehousing response was performed in all animals (N = 45). After rehousing, the systems and belts were removed from each animal and data from the recording devices were transferred via USB connection to a computer (software eMotion LAB, version 1.2.3.4, Mega Electronics, Kuopio, Finland).

HR and HRV were analyzed with the Kubios HRV software (version 2.1, Biomedical Analysis and Medical Imaging Group, Department of Applied Physics, University of Eastern Finland, Kuopio, Finland). Repeated short-term 5 min windows were analyzed [16]. To evaluate the stress around feeding, six time windows one day before rehousing were selected: during resting at 05.00 a.m., during time when light was switched on in the calf barn at approximately 06.40 a.m., 15 min before feeding in the morning, during feeding at approximately 07.20 a.m., 15 min after feeding and one hour after feeding. For the evaluation of the stress during rehousing four time windows were chosen: during resting at 05.00 a.m., during the rehousing process, 30 min after rehousing and one hour after rehousing.

Data were detrended (detrending method: smooth priors) and an artifact correction (level: 300 ms) was conducted. HRV variables in the time and frequency domain were analyzed as follows: HR, square root of variance of all R-R intervals (SDNN), root mean square of successive interbeat interval differences (RMSSD) as well as low-frequency power (LF; 0.04 to 0.3), high-frequency power (HF; 0.3 to 0.8 Hz), and the LF:HF ratio, whereby LF and HF were calculated by an autoregressive model and in normalized units (nu). The limits of the respiratory frequencies in calves were adopted from Mohr et al. [14]. Furthermore, short-term variability (SD 1), in a Poincaré plot, was analyzed.

### 2.4. Statistical Evaluation

All statistical analyses were performed using SAS (Version 9.3, SAS Institute Inc., Cary, NC, USA). To test the significance of the fixed effects, the GLIMMIX procedure in SAS was applied.

For analyses of milk intake (daily intake and weekly means) and body weight the feeding group (ad libitum, ADL; restricted, RES), sex (male and female) and day of life or week of life, respectively, were included as fixed effects. Calf within group was considered as random effect to account for repeated measurements. For weight at week 3 and daily weight gain, birth weight was included as covariate.

To ensure data quality, all HRV variables were screened for statistical outliers within each calf and time point using Hampel filtering. For each parameter, the median and the median absolute deviation (MAD) were calculated, and observations exceeding ±3 × 1.4826 × MAD from the median were classified as artifacts and excluded from further analysis. The constant 1.4826 scales the MAD to be comparable with one standard deviation of a normal distribution, providing a robust criterion for detecting outliers without imposing arbitrary physiological cut-off limits. For HR and HRV analyses, the feeding group, sex, time window, and the interaction of group × time window were included as fixed effects in the GLIMMIX model, with the individual calf considered as a random effect.

For all GLIMMIX models, residual covariance structures were compared, and an unstructured covariance matrix provided the best fit. Least square means were estimated for all fixed effects, and pairwise comparisons were performed using Tukey’s adjustment. Probability values less than 0.05 were considered statistically significant. Data are presented as LS-means (± standard error).

The number of animals included in this study was determined by the availability of newborn calves on the research farm. Therefore, no a priori power calculation was conducted. Nevertheless, the sample size (*n* = 39 for feeding analyses; *n* = 45 for rehousing analyses) was comparable to or larger than that used in similar physiological studies on calves [9,14,17,18]. To further indicate the robustness of the results, we additionally report exact *p*-values and standard errors for all fixed effects.

## 3. Results

### 3.1. Milk Intake and Body Weight

Until the third day of life, no difference in colostrum and transition milk intake was observed between both groups.

**Figure 1:**
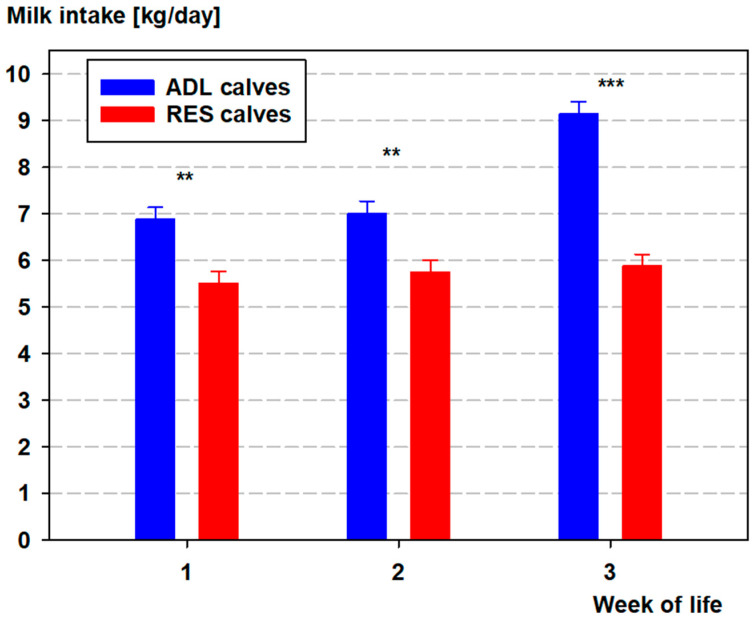
Weekly mean (LS-means ± SE) of daily milk intake (kg/day) of Holstein–Friesian calves fed ad libitum (ADL; *n* = 19, blue) or restrictively (RES; *n* = 20, red) during the first three weeks of life. Asterisks indicate significant group differences (** *p* < 0.05; *** *p* < 0.001).

Between d 4 and 7 of life, ADL calves ingested more milk in comparison with RES calves resulting in a higher intake on average during week 1 of life (Figure 1), but no differences were observed between d 8 and 10 of life. From d 10 to d 21 of life, ADL calves displayed higher daily intakes (*p* < 0.05 for each day). Two days before rehousing, the milk intake was higher in ADL calves (9.3 ± 0.39 L milk/d) in comparison with RES calves (5.8 ± 0.4 L milk/d; *p* < 0.05). Total milk consumption during the first three weeks of life was almost 35% higher in ADL calves (162 vs. 120 L milk/d; *p* < 0.05). In ADL calves, the sex of the animals had an effect on milk intake. Male calves (8.4 ± 0.12 L milk/d) displayed a higher daily milk intake compared with female calves (7.0 ± 0.13 L milk/d; *p* < 0.05). In RES calves, milk intake did not differ between male and female calves (5.7 ± 0.11 L milk/d vs. 5.7 ± 0.11 L milk/d; *p* = 0.9987).

Birth weights did not differ between ADL and RES calves (42.3 ± 1.3 kg vs. 42.4 ± 1.2 kg, *p* = 1.0). Body weight in both groups increased during the first three weeks of life, but were significantly higher in ADL calves in comparison to RES calves (58.7 ± 1.2 kg vs. 54.0 ± 1.1 kg, *p* < 0.05). In total, the ADL calves displayed a higher average daily gain between birth and d 23 of life (752 ± 56 g vs. 546 ± 53 g; *p* < 0.05). No differences were observed in average weights and daily weight gains between male and female animals, except for higher birth weights in RES male calves in comparison to RES female calves (Table 1).

### 3.2. HR and HRV Before, During, and After Feeding

**Figure 2:**
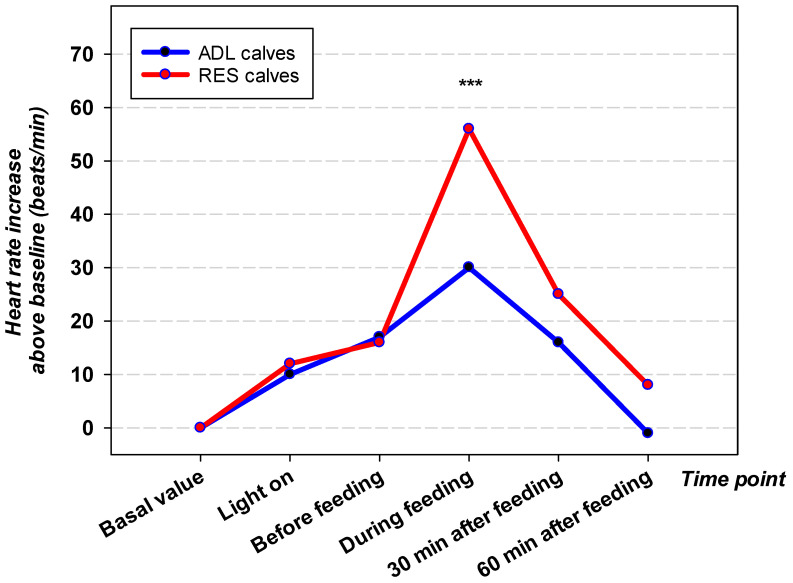
Change in heart rate (beats/min, base value subtracted) of ad libitum (ADL, N = 19) and restricted (RES, N = 20) milk-fed calves at different time points relative to feeding on d 21. Asterisks indicate significant differences between groups (*** *p* < 0.001).

The time window of measurement and the group significantly affected HR (both *p* < 0.05). The resting HR at 05.00 a.m. in ADL calves was higher in comparison to RES calves (*p* < 0.05; Table 2 and Figure 2).

During times of activity of the stall personnel such as after turning on the light and during preparation of the milk 15 min before feeding, the HR increased in both groups, but HR was still higher in the ADL group (each *p* < 0.05). At feeding, HR did not differ between groups (*p* = 1.0).

The HR increased 25% in ADL calves and 59% in RES calves (both *p* < 0.05) from resting to feeding. After feeding, HR decreased in both groups and was comparable to resting values. The *p*-values obtained for SDNN and RMSSD did not differ between the six time windows and both groups. One hour after feeding, RES calves displayed higher SDNN and RMSSD values compared with values during feeding (both *p* < 0.05). LFnorm and HFnorm were affected by the time window and the interaction of group and time window (*p* < 0.05). LFnorm was higher in ADL calves (76.3 ± 4.3 nu) compared with RES calves (61.3 ± 4.3 nu) at 05.00 a.m. (*p* < 0.05). Thereafter, LFnorm increased during feeding in RES calves in comparison to the resting value by 27% (76.7 ± 4.1 nu; *p* < 0.05). The resting HFnorm at 05.00 a.m. was lower in ADL calves (23.7 ± 4.3 nu) compared with RES calves (38.7 ± 4.3 nu; *p* < 0.05). HFnorm decreased until feeding in RES calves (23.8 ± 4.6 nu; *p* < 0.05). Values after feeding were comparable to resting values. Turning on the light in the calf barn particularly affected the LF:HF ratio in ADL calves which increased in comparison to resting values and which resulted in higher LF:HF ratios in comparison with the values of RES calves (*p* < 0.05). After the light was switched on, LF:HF decreased in ADL calves continuously until one hour after feeding. The parameter of the nonlinear domain SD1 displayed no differences between groups and time windows. SD1 decreased from 05.00 a.m. until feeding in RES calves and increased thereafter to the resting value.

### 3.3. HRV During Rehousing

The time window of measurement influenced all analyzed parameters of the rehousing process (each *p* < 0.05). Furthermore, the group affected HR and RMSSD as well as SD1, which differed between male and female animals (each *p* < 0.05). The results of all measured parameters of HRV at 05.00 a.m. were comparable between rehousing and feeding (Table 2 and Table 3). HR in ADL calves was higher compared with HR of RES calves before rehousing and 30 min after rehousing (both *p* < 0.05; Table 3). HR increased in both groups until rehousing and decreased thereafter. One hour after rehousing, the HR values in ADL calves were comparable to values one hour before rehousing, but in RES calves, values were still higher. The parameter SDNN did not differ between groups at any time window. From resting to one hour before rehousing, SDNN increased in RES calves (+394%, *p* < 0.05) and decreased thereafter. RMSSD values were 89% higher in ADL calves during rehousing compared with resting values (*p* < 0.05). In RES calves, rehousing and adaptation to the group pen resulted in lower RMSSD values in comparison to values during the rehousing process. LFnorm in RES calves increased by 28% after a 30 min period in the group pen in comparison to resting values (*p* < 0.05). The increase in LFnorm was reflected by a 28% decrease in HFnorm from resting to 30 min after rehousing in RES calves. The LF:HF ratio differed between both groups one hour before rehousing (53 ± 10 vs. 15 ± 9.5 for ADL and RES calves, respectively; *p* < 0.05). At 05.00 a.m., LF:HF was lower in ADL calves, but not in RES calves in comparison to values before rehousing. Thereafter, values in ADL calves decreased to levels which were comparable to resting values. The parameter of the nonlinear domain SD1 displayed no group differences at any time window. In ADL calves, SD1 increased from resting until rehousing and decreased thereafter.

Difference in sex of calves in HRV values were found for HR with a higher HR in male calves (132.4 ± 4.5 bpm) in comparison to female calves (112.3 ± 4.6 bpm; *p* < 0.01).

## 4. Discussion

This study was performed to investigate the effect of a restrictive feeding protocol in comparison to an ad libitum supply of whole milk during the neonatal period from birth to week 3 of life on the cardiac stress response of dairy calves. The feeding intensity of the RES calves followed the standard protocol of 10% of BW per day, which remains commonly practiced in dairy and beef calf rearing in Germany and is comparable to protocols applied in the US. The mean uptake of acidified milk by the ADL calves of approximately 8 L per day was consistent with previous reports [2,3], although slightly higher intakes of ~10–11 L/day have also been described in male and female calves [1,19]. In our study, milk intake in ADL calves decreased to RES levels between d 8 and 11, most likely due to the late change from transition to acidified milk on d 5, which is known to reduce acceptance. Nevertheless, before HR and HRV measurements were conducted, ADL calves had consumed substantially more milk for >10 days, so differences in cardiac responses can be attributed to feeding intensity.

The observed group differences in HR and HRV likely reflect underlying shifts in autonomic balance. Restricted-fed calves exhibited lower resting HR and LF:HF ratio, suggesting parasympathetic predominance at rest, which is typically associated with energy conservation under nutritional limitation. During feeding, the pronounced increase in HR and LF power together with a decline in HF power indicate activation of the sympathetic nervous system and withdrawal of vagal tone, consistent with a physiological arousal response driven by hunger and feeding motivation. In contrast, ad libitum calves maintained a more balanced sympathetic–parasympathetic activity, implying that sufficient nutrient supply reduced the need for autonomic adjustment. This pattern aligns with the general concept that energy restriction enhances sympathetic responsiveness and reduces parasympathetic stability in mammals.

On dairy farms, calves are typically exposed to various potential environmental- and management-related stressors. The autonomous cardiac stress reaction of the individual animal to such stressors depends on various factors such as on previous positive or negative experiences and on the level of psycho-physiological and mental stress [12,13,20]. In addition, nutritional supply has been shown to affect the autonomic cardiac response in both humans and animals [21]. In healthy young women, fasting from 12 to 48 h has been associated with changes in HRV parameters, reflected by a lower heart rate, a reduced LF component, and an increased HF component [22,23]. These findings demonstrate that insufficient nutritional supply in humans is consistently associated with autonomic adjustments characteristic of a physiological hunger state, which are comparable to those described in feed-restricted farm animals.

Physiological resting HR in Holstein–Friesian calves declines with age but generally remains above 100 beats/min throughout the pre-weaning period. In one Slovakian study, values of around 160 beats/min were reported within two hours after birth [17], while another study conducted in Brazil observed 137 beats/min at day 21 [24], and further work in Germany reported ~105 ± 11 beats/min at weaning [14]. Values close to 100 beats/min were also described in 14 or 15-day-old calves that were individually housed and received only 6 L of milk per day [18,25], suggesting that restricted feeding intensity can reduce HR. Against this background, the value of 96 beats/min observed in our RES calves at day 21 during resting appears unusually low and may indicate a latent hunger state caused by limited milk allowance. Comparable effects of reduced energy intake on HR have been reported in other species. In neonatal rats, brief maternal separation induces bradycardia that normalizes with feeding [26], while chronic calorie restriction (30–40% of ad libitum intake) significantly lowers HR and recovers promptly when energy intake is restored in adult rats [27]. These effects are mediated by shifts in sympathetic and vagal tone as well as beta- and alpha-adrenergic pathways. In line with this, studies in cattle, young goats, and broiler chickens report higher resting HR in ad libitum-fed systems compared with restrictively fed animals [28,29,30]. Together, these findings underline that energy intake is a key determinant of cardiac autonomic regulation across species and support the interpretation that restricted milk feeding placed our calves in a physiological state of hunger. Since HR can also indicate energy status, a higher energy expenditure in ADL calves can be assumed [31].

Feeding time and rehousing were chosen as representative stress-inducing scenarios. Restrictively fed calves are often restless and vocalize when personnel enter the barn, consistent with reports that they consume milk quickly without satiety [32] and show more non-nutritive sucking [3,7]. In our study, ADL calves also stood before feeding but did not vocalize, suggesting that restlessness of RES calves may have spread socially. Rehousing calves from individual hutches into group pens is a common procedure. Although the brief weighing immediately beforehand likely added to the stress response, it was identical for all calves and therefore does not confound group comparisons. All calves were unfamiliar with being haltered and walked, and general activity increased after rehousing, as previously described [33]. Animals also started to sniff and explore each other soon after. Therefore, the HRV could only be analyzed for one hour after rehousing in the unobserved animals.

During the trial, the HRV recording system was well tolerated. Because activity of the calves in their hutches caused movement of the electrodes in some cases, only 5 min periods with regular recordings were analyzed. To evaluate the resting values, video recordings of 15 animals of each group were analyzed. At 05.00 a.m. all animals were lying and activity of the other calves at this time period was not expected, but cannot be completely ruled out. HR and HRV values were largely comparable to previous studies and confirmed stress induction by both feeding and rehousing [14,34]. Interestingly, ADL calves also showed increased HR during feeding. As the study was carried out during wintertime, and according to our observation with increasing age, calves drank increasing amounts of milk within the first hour after feeding. Calves seemed to prefer the warm milk and learned to wait for the next feeding period, but this was not associated with higher diarrhea prevalence. Importantly, the relative HR increase during feeding and rehousing was greater in RES calves, consistent with stronger sympathetic activation. The cardiac stress response to feeding activity reflected by HR was only short because one hour after fresh milk supply, the HR was comparable to resting values. Also, the speed of the individual calves during the walk of the 200 m distance throughout the rehousing process differed, which might be reflected by a variance within the HR. In contrast to these findings, it can be assumed that the adaptation of the calves to rehousing took more than one hour because HR was still higher at that time in comparison to resting values.

Interpretation of HRV parameters provides additional insights. Time domain analysis revealed that SDNN was lowest in RES calves during feeding but did not differ between groups during rehousing. Both RMSSD and SDNN were lowest during feeding in RES calves and after rehousing in both groups, consistent with parasympathetic withdrawal under stress [14,35]. Unexpectedly, ADL calves showed a transient increase in RMSSD and SD1 during rehousing, suggesting vagal activation, though values soon declined [36,37]. Frequency domain analysis showed increased LF power in RES calves during feeding, indicating a sympatho-vagal shift towards sympathetic dominance [36,38,39,40], while HF power decreased, reflecting reduced vagal tone [13,16,36,41]. LF:HF ratios increased in both groups when light was switched on and before rehousing. The peak at light-on in ADL calves likely reflects a startle response to the sudden illumination, as confirmed by video recordings of personnel switching on the light. Such a peak was not observed in RES calves, which may have been more alert in anticipation of feeding due to hearing the approaching personnel, so that the additional stimulus of light was less pronounced.

Taken together, HR and HRV measures indicate that restricted milk feeding increases stress load around feeding, evident from behavioral restlessness and a shift towards sympathetic dominance. Responses to rehousing were less conclusive, with some unexpected HRV patterns in ADL calves that warrant further study. Our findings support the use of HR and HRV to assess acute stress in calves, but also highlight limitations. Comparisons between calf studies remain difficult due to missing standardization of HRV analysis [16]. Future work should include plasma catecholamines to validate autonomic activation [42] and explore nonlinear HRV metrics that can differentiate stress intensity more precisely than linear parameters [14].

## 5. Conclusions

Restrictive milk feeding in neonatal calves was associated with altered cardiac autonomic regulation, reflected by lower heart rate and reduced LF:HF ratio during resting compared to ad libitum-fed calves. These results suggest that restricted calves may have experienced a physiological state comparable to hunger, while ad libitum feeding was linked to higher energy expenditure. Feeding and rehousing both induced short-term stress responses, with more pronounced sympathetic activation in restrictively fed animals. Our findings demonstrate that heart rate and heart rate variability are valuable non-invasive welfare assessment indicators in calves and highlight the need for further research using standardized HRV analyses and complementary physiological measures. From a practical perspective, providing calves with unrestricted milk access may help to reduce physiological stress and improve welfare in early rearing systems.

## Figures and Tables

**Table 1:** Weight parameters [LS-means ± SE] of calves fed ad libitum (ADL) and restrictively (RES) during the first three weeks of life. Different superscripts (a, b) within a row indicate significant differences (*p* < 0.05).

Parameter	ADL Male	ADL Female	RES Male	RES Female
**Birth weight, kg**	42.1 ± 1.4 ^a,b^	43.1 ± 1.3 ^a,b^	45.5 ± 1.4 ^b^	39.6 ± 1.3 ^a^
**Weight on d 23, kg**	57.6 ± 1.8 ^a,b^	59.8 ± 1.6 ^a^	54.9 ± 1.7 ^a,b^	53.2 ± 1.6 ^b^
**Ø daily weight gain, d 1–23, g/d**	725 ± 83	778 ± 76	568 ± 81	524 ± 77

**Table 2:** Heart rate (HR) and heart rate variability (HRV) parameters [least squares means ± SE] of Holstein–Friesian calves fed either ad libitum (ADL; 9 male and 10 female calves) or restrictively (RES; 8 male and 12 female calves) during six consecutive 5 min windows around morning feeding (day 21 ± 2 days). Superscripts (a–c) indicate significant differences (*p* < 0.05) of one parameter across different sampling times within the same group (row-wise). Bold values denote significant differences (*p* < 0.05) between groups within the same time window.

Parameter	Group	Resting Value	Activity of Personnel	Time Window in Relation to Feed Supply			
		05.00 a.m.	Light on	15 min Before	During	15 min After	1 h After
**HR (bpm)**	ADL	**120 ^b^**	**131 ^b^**	**137 ^ab^**	151 ^a^	**136 ^ab^**	**119 ^b^**
	RES	**96 ^c^**	**108 ^bc^**	**112 ^bc^**	152 ^a^	**121 ^b^**	**103 ^c^**
**SDNN (ms)**	ADL	41.4	46.7	41.2	35.5	34.2	39.9
	RES	35.3 ^ab^	41.4 ^ab^	37.9 ^ab^	27.4 ^b^	43.1 ^ab^	47.9 ^a^
**RMSSD (ms)**	ADL	40.2	39.4	33.0	34.4	36.4	37.7
	RES	41.2	37.5	38.9	35.8	39.4	39.9
**LF_norm_ (nu)**	ADL	**76.3 ^ab^**	81.9 ^a^	78.2 ^ab^	79.4 ^ab^	69.3 ^ab^	65.1 ^b^
	RES	**61.3 ^b^**	76.7 ^a^	73.6 ^ab^	77.9 ^ab^	76.1 ^ab^	67.9 ^ab^
**HF_norm_ (nu)**	ADL	**23.7 ^ab^**	18.1 ^b^	21.7 ^ab^	20.6 ^ab^	30.7 ^ab^	34.8 ^a^
	RES	**38.7 ^a^**	23.3 ^b^	26.4 ^ab^	23.8 ^ab^	29.2 ^ab^	32.0 ^ab^
**LF:HF ratio**	ADL	7.7 ^b^	**28.1 ^a^**	5.6 ^b^	7.4 ^b^	8.7 ^b^	2.3 ^b^
	RES	2.2	**7.7**	4.1	6.9	5.0	5.8
**SD1 (ms)**	ADL	28.5	27.9	23.3	24.4	25.8	26.6
	RES	29.1	26.5	27.5	25.4	27.9	28.2

SDNN: square root of variance of all R-R intervals, RMSSD: root mean square of successive interbeat interval differences, LF: low-frequency power, HF: high-frequency power, SD1: standard deviation of instantaneous HRV measured from axis 1 in Poincaré plot.

**Table 3:** Heart rate (HR) and heart rate variability (HRV) parameters [least squares means ± SE] of Holstein–Friesian calves fed either ad libitum (ADL; 9 male and 12 female calves) or restrictively (RES; 12 male and 12 female calves) before, during, and after rehousing (mean age 22.7 ± 2 days). Superscripts (a–d) indicate significant differences (*p* < 0.05) of one parameter across different sampling times within the same group (row-wise). Bold values denote significant differences (*p* < 0.05) between groups within the same time window.

Parameter	Group	Resting Value	Time Window in Relation to Rehousing			
		05.00 a.m.	1 H Before	During	30 min After	1 h After
HR (bpm)	ADL	**122 ^d^**	**127 ^cd^**	177 ^a^	**155 ^b^**	143 ^cb^
	RES	**98 ^c^**	**111 ^c^**	171 ^a^	**138 ^b^**	130 ^b^
SDNN (ms)	ADL	29.3	92.6	71.1	52.5	35.1
	RES	30.6 ^b^	151.0 ^a^	70.3 ^ab^	47.9 ^b^	58.3 ^ab^
RMSSD (ms)	ADL	31.3 ^b^	50.7 ^ab^	59.2^a^	36.5 ^ab^	26.2 ^b^
	RES	40.0 ^abc^	56.6 ^ab^	57.0^a^	28.9 ^c^	34.6 ^bc^
LF_norm_ (nu)	ADL	**76.0**	70.5	65.2	75.7	76.4
	RES	**59.9 ^b^**	71.3 ^ab^	64.1 ^ab^	76.7 ^a^	75.2 ^ab^
HF_norm_ (nu)	ADL	**24.0**	29.4	34.7	24.2	23.6
	RES	**40.1 ^a^**	28.7 ^ab^	35.8 ^ab^	23.2 ^b^	24.8 ^ab^
LF:HF ratio	ADL	7.0	8.0	2.9	11.2	12.8
	RES	1.5	15.4	2.1	11.7	17.9
SD1 (ms)	ADL	22.1 ^b^	35.9 ^ab^	41.9 ^a^	25.8 ^ab^	18.5 ^b^
	RES	28.3 ^ab^	40.3 ^a^	40.3 ^a^	20.4 ^b^	24.5 ^ab^

SDNN: square root of variance of all R-R intervals, RMSSD: root mean square of successive interbeat interval differences, LF: low-frequency power, HF: high-frequency power, SD1: standard deviation of instantaneous HRV measured from axis 1 in Poincaré plot.

## Data Availability

The original contributions presented in this study are included in the article material. Further inquiries can be directed to the corresponding author.

## Abbreviations

The following abbreviations are used in this manuscript:

ADL	ad libitum-fed calves
RES	restrictively fed calves
HR	heart rate
HRV	heart rate variability
SDNN	square root of variance of all R-R intervals
RMSSD	root mean square of successive interbeat interval differences
LF	low-frequency power
HF	high-frequency power
SD1	standard deviation of instantaneous HRV measured from axis 1 in Poincaré plot
